# HLA Class I Restriction as a Possible Driving Force for Chikungunya Evolution

**DOI:** 10.1371/journal.pone.0009291

**Published:** 2010-02-26

**Authors:** Joo Chuan Tong, Diane Simarmata, Raymond T. P. Lin, Laurent Rénia, Lisa F. P. Ng

**Affiliations:** 1 Data Mining Department, Institute for Infocomm Research, Fusionopolis, Singapore, Singapore; 2 Department of Biochemistry, Yong Loo School of Medicine, National University of Singapore, Singapore, Singapore; 3 Singapore Immunology Network, Biopolis, Singapore, Singapore; 4 National Public Health Laboratory, Communicable Diseases Division, Ministry of Health, Singapore, Singapore; Université Pierre et Marie Curie, France

## Abstract

After two decades of quiescence, epidemic resurgence of Chikungunya fever (CHIKF) was reported in Africa, several islands in the Indian Ocean, South-East Asia and the Pacific causing unprecedented morbidity with some cases of fatality. Early phylogenetic analyses based on partial sequences of Chikungunya virus (CHIKV) have led to speculation that the virus behind recent epidemics may result in greater pathogenicity. To understand the reasons for these new epidemics, we first performed extensive analyses of existing CHIKV sequences from its introduction in 1952 to 2009. Our results revealed the existence of a continuous genotypic lineage, suggesting selective pressure is active in CHIKV evolution. We further showed that CHIKV is undergoing mild positive selection, and that site-specific mutations may be driven by cell-mediated immune pressure, with occasional changes that resulted in the loss of human leukocyte antigen (HLA) class I-restricting elements. These findings provide a basis to understand Chikungunya virus evolution and reveal the power of post-genomic analyses to understand CHIKV and other viral epidemiology. Such an approach is useful for studying the impact of host immunity on pathogen evolution, and may help identify appropriate antigens suitable for subunit vaccine formulations.

## Introduction

Chikungunya virus (CHIKV), an *Alphavirus* belonging to the *Togaviridae* family, was first isolated during a Tanzanian (formerly Tanganyika) outbreak in 1952 [1], [2]. Between 1960s–80s, the pathogen infected more than 100,000 people in Africa and Asia, before entering a state of quiescence for over two decades [3]–[5]. In recent years, CHIKV has re-emerged as one of the major important infections in South-East Asia and the Pacific region, causing considerable morbidity with even some cases of fatality [6]. Epidemic resurgence of disease was reported in the Democratic Republic of Congo in 2000 [7], in Indonesia during 2001–03 [8], in India during 2005–06 [9], in Malaysia in 2006 [10], and in Singapore in 2008 [11]. During the same period, the virus was also isolated in several islands of the Indian Ocean, including Réunion Island, Maldives, Mayotte, Mauritius and Seychelles [12]. Typical clinical presentations of CHIKF include fever, headache, nausea, vomiting, myalgia, rash and arthralgia, and can be accompanied by severe, debilitating joint pain lasting from months to years [13]–[15]. Recent outbreaks of CHIKF involving millions of people have resulted in more detailed descriptions of clinical manifestations, including rare or previously unknown complications, such as fatal haemorrhagic and neurologic manifestations [16], [17]. How and why CHIKV resurfaced after an interval of more than twenty years remains unknown. The ability to replicate more efficiently in another mosquito vector, *Aedes albopictus*, has been proposed as a factor for the new epidemics [18].

In many viral infections, the adaptive immune system has been shown to exert a strong pressure on viral evolution. In particular, antigenic drift, a phenomenon observed for Influenza virus neuraminidase and hemagglutinin, was shown to result from the accumulation of mutations in viral sequences recognized by antibodies [19]. In many viral infections, escape mutants in the face of CD8^+^ cytotoxic T cells has been widely described, demonstrating an important role for the major histocompatibility complex (MHC). CD8^+^ T lymphocytes recognize short sequences in proteins, termed T-cell epitopes, cleaved from viral proteins and presented on the surface of infected cells by HLA class I molecules, also called HLA in humans. HLA molecules are highly polymorphic and can only present viral epitopes that have the appropriate amino acid composition to enable binding. We hypothesized that mutation patterns in CD8^+^ epitopes may provide important clues to what kind of immune selection pressure operates on CHIKV. The viral sequence is, therefore, expected to show patterns of mutations in epitope sequences presented by MHC class I molecules. In particular, the rapid emergence of sequence variation within T-cell epitopes provides clear evidence for host-driven immune selection during infection [20], [21].

Phylogenetic analyses based on partial envelope glycoprotein (E) 1 sequences have revealed important insights into the evolution of CHIKV, suggesting the existence of three distinct phylogroups: one from Asia, one from Western Africa, and one from Eastern, Central and Southern Africa [13]. In this study, we extended the analysis to full CHIKV structural and non-structural sequences and examined for the first time, the genetic diversity and antigenic relationships of CHIKV sequences from its introduction into humans in 1952 to 2009 to assess the extent of geographical variability and existence of potential selective pressure. We employed post-genomic approaches to identify the basis of CHIKV resurgence and aimed to show that site-specific mutations and variations may be driven by cell-mediated immune pressure, with occasional changes that resulted in the huge loss of human leukocyte antigen (HLA) class I-restricting elements. These findings may provide an explanation for the explosive viral outbreaks observed since 2005.

## Results and Discussion

**Figure 1:**
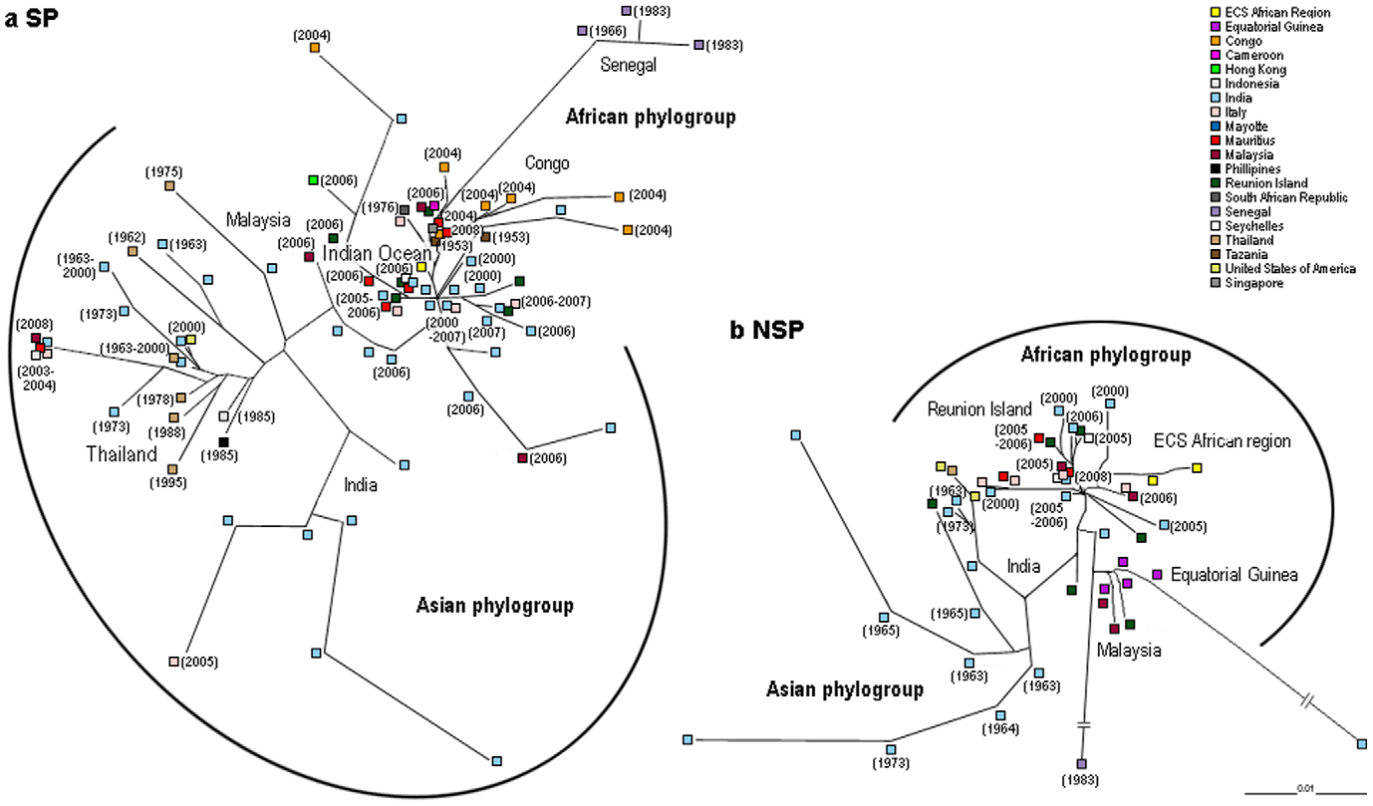
Phylogenetic relationships among CHIKV isolates. **a.** Structural polyproteins and **b.** Non-structural polyproteins based on full nucleotide sequences. CHIKV is a linear, positive-sense, single-stranded RNA genome of approximately 12,000 nucleotides. The genome contains two large open reading frames (ORF) encoding the non-structural polyprotein (nsP) (2,474 aa) and structural polyprotein (sP) (1,248 aa) respectively. ORF1 encodes non-structural proteins nsP1 (535 aa), nsP2 (798 aa), nsP3 (530 aa) and nsP4 (611 aa). ORF2 encodes structural proteins, including one capsid protein (C), two major envelope surface glycoproteins (E1, E2) and two small proteins (E3, 6K). The branches leading to the Senegal strain and the India strain were shortened by 40% for convenience.

First, we examined the genetic diversity and antigenic relationships of CHIKV sequences from its introduction into humans in 1952 to 2009 to assess the extent of geographical variability and existence of potential selective pressure. Previous studies based on partial E1 sequences have shown the existence of three distinct CHIKV genotypes: one from Asia, one from Western Africa, and one from Eastern, Central and Southern (ECS) Africa [13]. Complete phylogenetic analysis of full CHIKV structural and non-structural sequences was performed to appreciate the extent of CHIKV proteome variation. Our results showed the existence of two main phylogroups: one from Asia and one from Africa (Figure 1). The Asian, ECS African, and Western African genotypes were divergent. The observed pattern of phylogenetic structure is consistent with existing studies based on CHIKV E1 protein sequences [13], [22]. For both structural and non-structural sequences, the Indian Ocean isolates represent a homogenous clade within the India isolates, and form a continuous lineage; from the Asian cluster, through the Indian Ocean cluster, to the African cluster.

**Figure 2:**
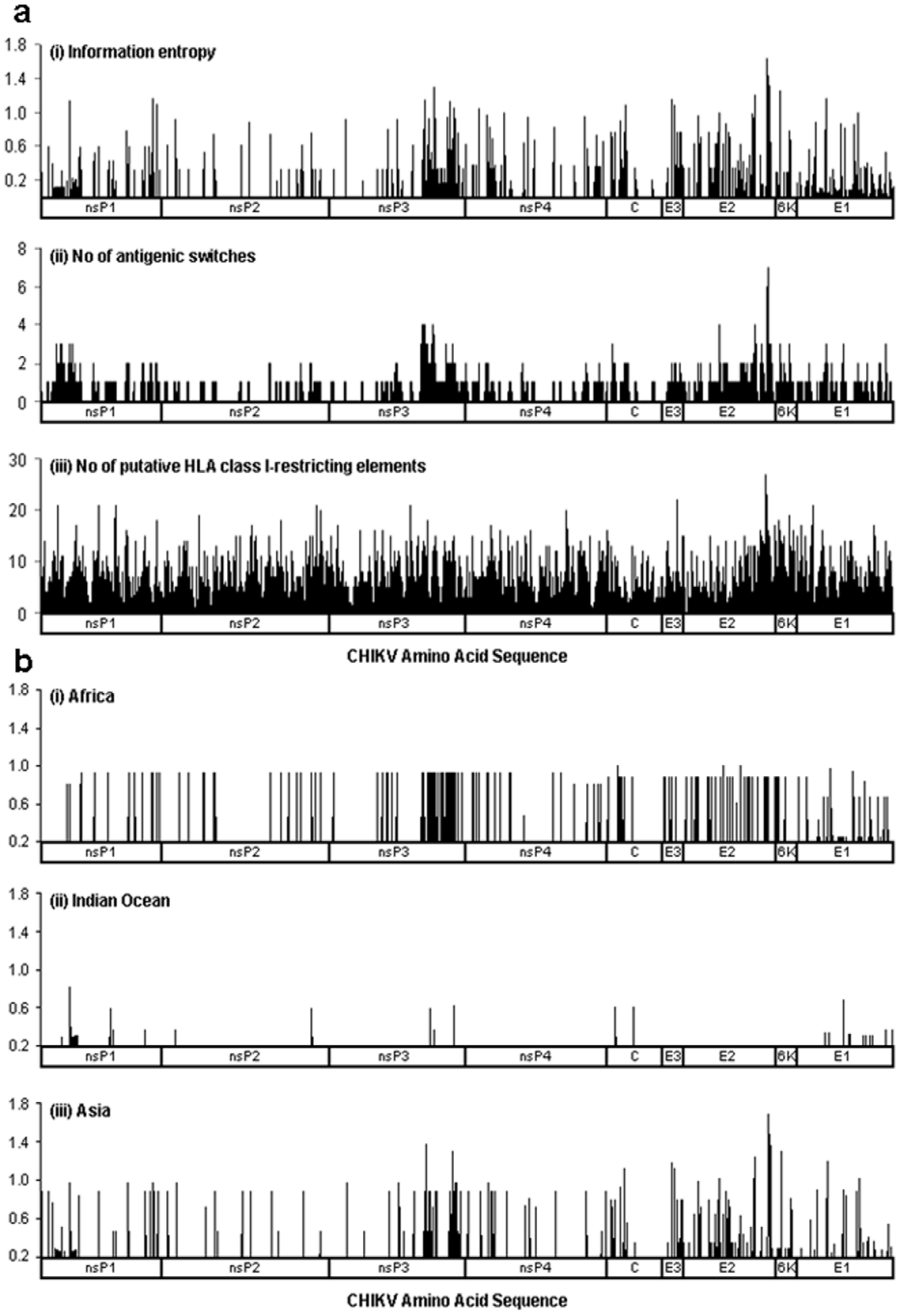
Position-specific plots of CHIKV amino acid sequences from 1952 to 2008. **a.** Overview of all CHIKV amino acid sequences. i. Entropy profile. Sequence variability is observed at almost every position (entropy >0.1) in E1 and E2. The E3, 6k and C-terminal region of nsP3 also demonstrated a history of sequence variability. ii. Profile illustrating the loss of HLA class-I restricting elements due to CHIKV position-specific mutations, and iii. Profile illustrating the number of putative HLA class I-restricting elements at each position along the CHIKV primary sequence. **b.** Entropy profiles of CHIKV sequences. i. Africa, ii. Indian Ocean, and iii. Asia.

We next applied position-specific plots to examine the extent of amino acid conservation in the CHIKV genome. This can also reveal the spatial dynamics of mutations at any specific positions. We define an “antigenic switch” as the change in expression of CHIKV genes at a specific site which may 1) abrogate binding to HLA molecule [23]; or 2) antagonize or interfere T-cell response leading to cellular immune evasion [24], [25] (Figure S1). Our analysis revealed that significant amounts of antigenic switches were clustered over the CHIKV genome (Figure 2a). In particular, residues 697–709 of E2 structural protein have undergone the most number of substitutions with up to seven site-specific mutations in isolates derived from India, Mauritius and Senegal since 1983. Recent studies have shown that this domain is important for vector infectivity of CHIKV and might also play an epistatic role in adaptation of the virus to *Ae. Albopictus* and *Ae. Aegypti* [26]. On the contrary, the non-structural proteins nsP1, nsP2, the N-terminal region of non-structural protein (nsP) 3, nsP4, as well as the structural region of capsid protein (C), contained many historically fully conserved regions (entropy  = 0.0) (Figure 2a and b). No amino acid frequency changes were observed at nsP2, nsP4, C, E3, E2 and 6K for CHIKV Indian Ocean isolates, except four substitutions (nsP2-Y643N, C-C54R, C-D132N and E2-Q471R) between Mauritius isolate Wuerzburg [27], Réunion isolates 06.21, 06.27 [13] and Seychelles isolate 05.209 [13]. Deletions were found to occur primarily within the capsid domain at positions 83, 114 and 127.

**Figure 3:**
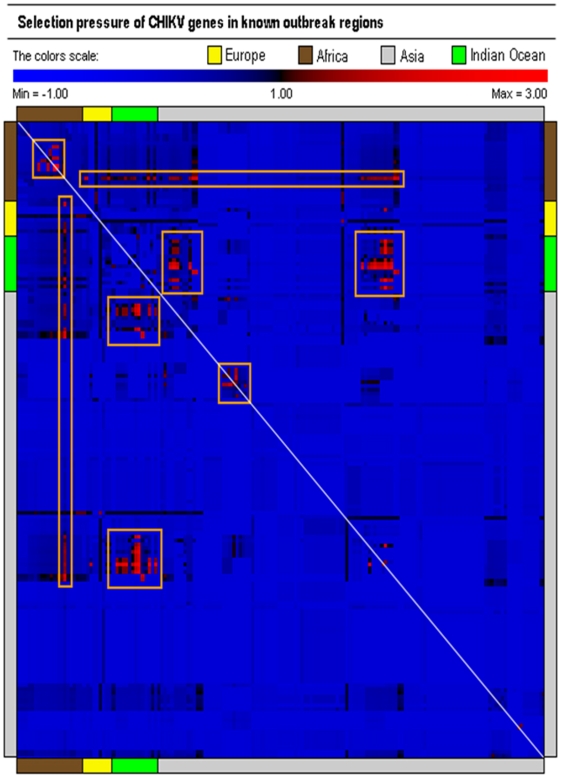
Proposed antigenic relationships among CHIKV isolates. The Nei and Gojobori's map showing the selection pressure of reported isolates in pairwise comparison of CHIKV nucleotide sequences. Blue indicates negative selection (*ω*<1), black for neutral substitutions (*ω* = 1) and red for positive selection (*ω*>1). Orange boxes are drawn around positive selection clusters.

**Figure 4:**
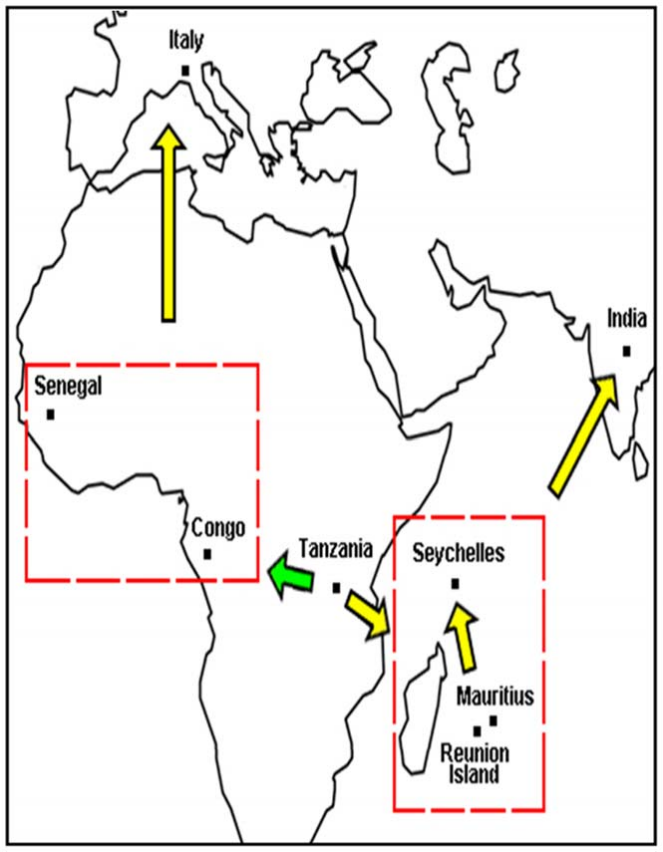
Proposed evolutionary scenario of CHIKV isolates. CHIKV evolution was proposed from its introduction in 1952 to 2008 based on positive selection pressure of *ω*>1 calculated using the Nei and Gojobori's [30], [31]. The dotted square indicates regions with possibly mixed CHIKV transmissions, while the green arrows indicate hypothetical evolutionary routes.

The rate of synonymous and non-synonymous nucleotide substitutions can be used as a basis for studying molecular sequence evolution [28]. We then applied the Nei and Gojobori's method [29], [30] to analyze the pairwise selection pressures of CHIKV nucleotide sequences (Figure 3). A small class of structural genes (167 out of 13530 pairs), with positive selection pressure of *ω*>1 was found. Among them, evidence for diversifying selection (*ω*>1) was observed in the structural genes of reported isolates derived from seven countries: Democratic Republic of Congo, Senegal, Reunion Island, Mauritius, Seychelles, India and Italy, all of which appear to be under mild positive selection (Figure 4). The India Chennai isolates 02TANUVAS and 04TANUVAS, with strength of selection *ω*≈1.52, shared 92.3% nucleotide identity. The E2 protein potential glycosylation sites at positions 263 and 345 were fully conserved [31]. Amino acid changes were found between protein positions 241–243, 346–349 and 369–376. Five substitutions were observed in the E1 proteins: Y123H, Y181C, L242V, S350N and Q352R. Among them, E1 proteins of the Democratic Republic of Congo isolates DRC1720 and DRC1725/28 (nucleotide identity 99.4%) are under the highest selection pressure, with *ω*≈2.92 (Figure 3 and 4).

**Figure 5:**
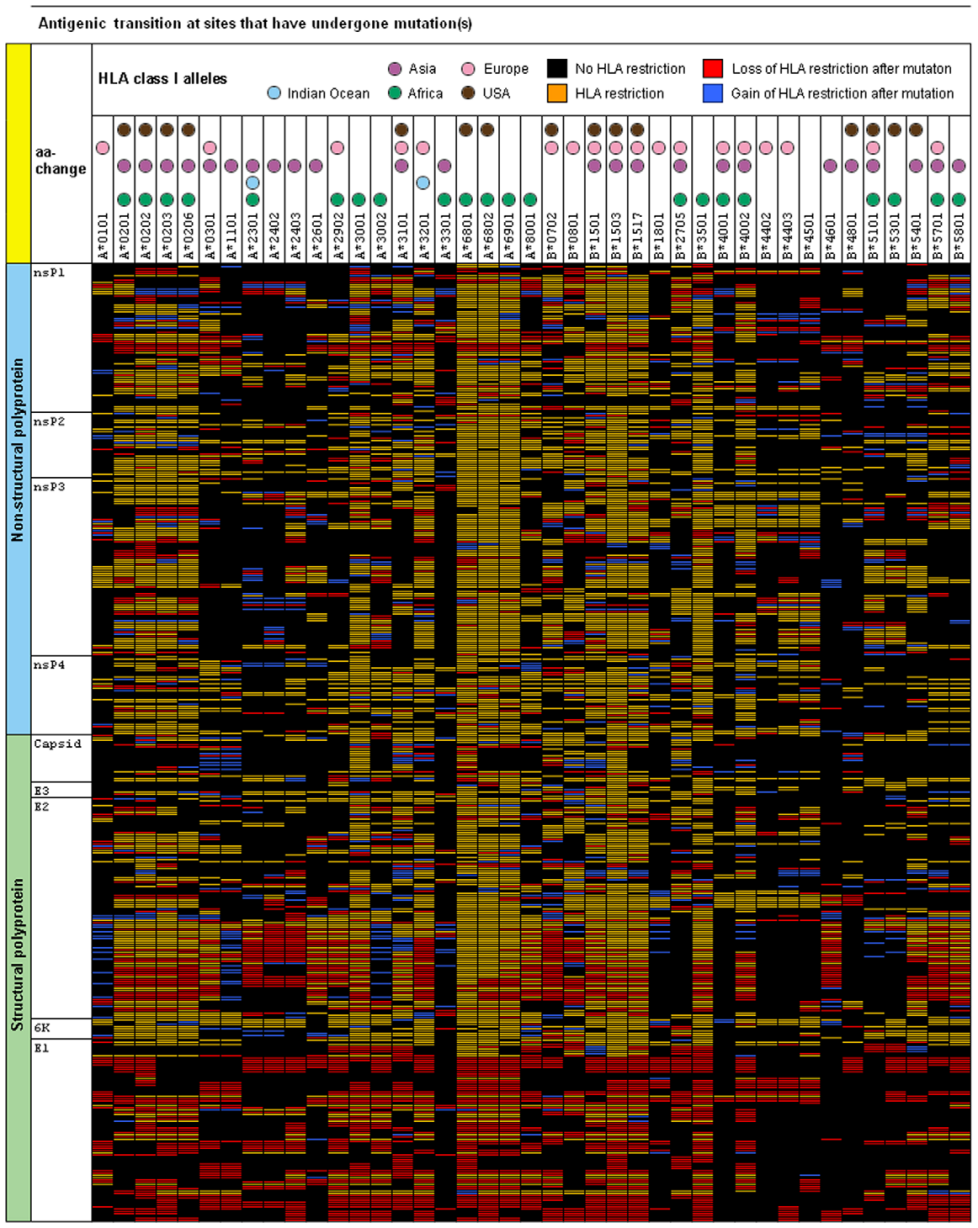
Putative antigenic transitions showing the change in HLA class I-restricted recognition patterns at sites that have undergone mutations. Significant changes in HLA class I-restricted recognition patterns are observed in E1 and E2. HLA alleles that are common in Africa, Indian Ocean, Asia, Europe and USA are indicated in colored circles. The correlation between amino acid and antigenic variability was 0.73 (nsP = 0.72, sP = 0.74), and on average, 5 amino acid substitutions resulted in one antigenic switch (s.d = 1.08).

Having shown that regions of the CHIKV were under selective pressure, we hypothesize that this pressure may be immune mediated, and in particular by HLA molecules. Thus, we decided to search for putative CD8^+^ T-cell epitopes restricted by 41 common HLA class I alleles. The T-cell epitope predictors used in this paper are derived from the Immune Epitope Database and Analysis Resource [32]. We used all 41 HLA class I predictors that are available at the time of study, focusing on nonameric peptide sequences, because they represent the predominant length of HLA class I-restricted T-cell epitopes [33]. As illustrated, we find remarkable overall correspondence between amino acid sequence and antigenic switch variability (correlation coefficient  = 0.73) (Figure 2a). Increased changes in amino acid contents result in a higher switch frequency. The rate of antigenic switches per substitution (i.e., the rate of change from HLA binding to non-binding peptide) was fastest within 6K and E2 domains (3.27 and 3.58 switches per amino acid respectively), and slowest within nsP2 and nsP4 domains (8.33 and 7.18 switches per amino acid respectively). We found significant changes in potential HLA class I-restricted recognition patterns within E1 and E2 domains that have undergone mutations (Figure 5). Among all substituted sites, only two mutations, nsP3-V1770A and E2-D457E appear fixed with no changes in HLA class I restricted recognition patterns. Strikingly, 62 substitutions resulted in the loss of restriction to all 41 putative HLA class I alleles, and multiple substitutions has helped enhanced the antigenic switch process (Figure 3 and 5). The correlation between antigenic switch frequency and the number of amino acid substitutions was 0.89. The observed patterns of antigenic transitions are most pronounced within the E1 domain, where 58 out of 81 antigenic switches resulted in the complete loss of restriction to all 41 HLA class I alleles. Mutations at these putative sites appear to be largely driven by selective immune evasion.

**Figure 6:**
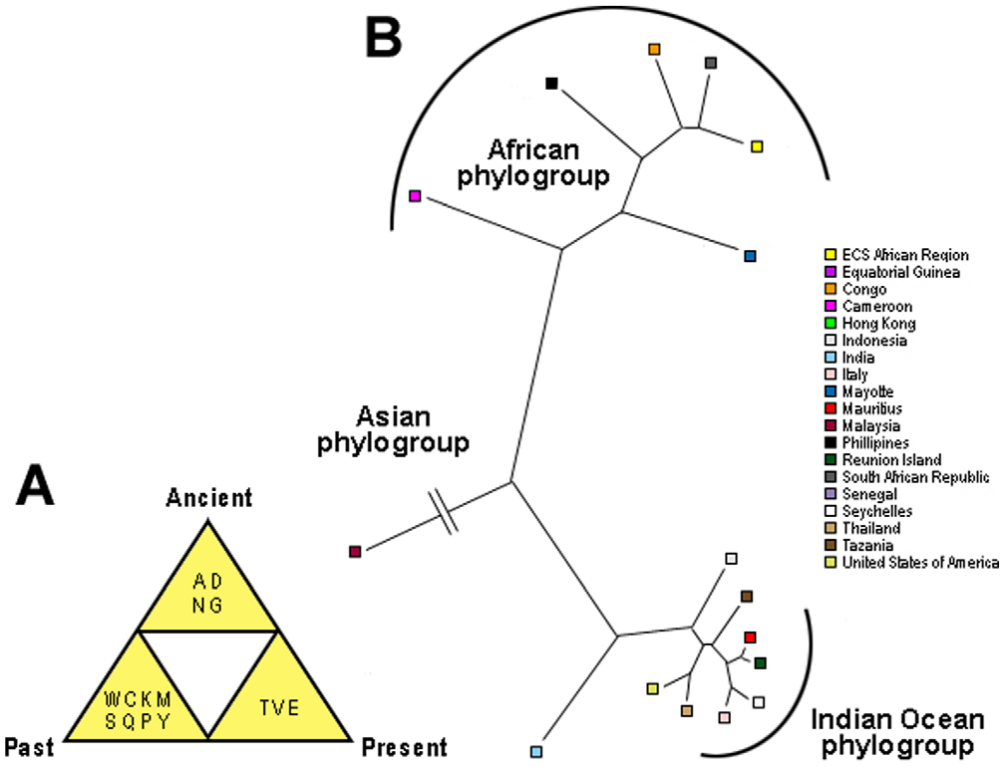
Immunological signatures among CHIKV isolates. **a.** Cluster-difference amino acid preferences, defined using three timelines: ancient (1950s–1970s), past (1980s–1990s) and present (2000s). **b.** Relationship between CHIKV immunological signatures. Immunological signatures from reported isolates in outbreak countries were obtained using the nearest neighbor algorithm. Three main clusters can be identified in this study: African, Asian and Indian Ocean phylogroups. The branch leading to Malaysian strain was shortened by 50% for convenience.

We identified a total of 6336 (nsP = 3931, sP = 2405) immunological signatures or unique HLA class I-restricted T-cell epitope candidates. There are 477 (nsP = 209, sP = 268) substitutions from 407 (nsP = 191, sP = 216) sites, resulting in an increased diversity of 668 immunological signatures. Across CHIKV outbreak regions, immunological signatures specific to ECS Africa (9/322), Congo (18/336), Senegal (310/930), Seychelles (9/1231), India (314/1554), Malaysia (12/223), and USA (27/1222) were observed among the structural genes. No immunological signatures unique to CHIKV outbreak regions are observed among the non-structural genes, suggesting the lack of selection pressure by HLA class I alleles on these genes. The ability to define unique signatures allows us to identify the amino acid compositions that characterize the difference between countries (Figure 6a and b). Some of these amino acid substitutions may lead to the antigenic difference between countries, some may be compensatory changes to retain function, and others may be hitchhikers that evolved purely by chance [34]. Consistent with phylogenetic analysis of CHIKV full structural and non-structural sequences, three distinct groups may be observed with respect to these immunological signatures: African, Indian Ocean and Asian phylogroups, with average correlations of 0.88, 0.89 and 0.86 respectively. The African phylogroup contains has a higher concentration of Ala (30%), Ser (20%) and Val (19%) compared to the Indian Ocean and Asian phylogroups. For the Indian Ocean phylogroup, a higher concentration of Lys (19%), Leu (30%), Pro (23%) and Arg (31%) was detected (Figure 6a and b). Collectively, these findings suggest that some amino acid substitutions may possess larger antigenic effect in certain geographical regions.

CHIKV is endemic in many parts of Africa and Asia with variable levels of transmission in populations which are largely immune [8], [35]–[37]. In naive populations, massive epidemics occur before herd immunity develop and curb virus dissemination in the population. Different extrinsic and intrinsic viral factors could variously be ascribed to continuous viral evolution leading to novel virulence and survival properties of CHIKV, such as i) changes in demography and human behaviour, and ii) sequence variation. The latter could result to a change of vector competence and vector ecology leading to increased transmission, and the decrease or absence of antibody or T cell recognition leading to less viral inhibition by the adaptive immune system.

Here, we employed post-genomic approaches to identify a possible basis for CHIKV resurgence. The availability of viral isolates and genetic sequences, coupled with bioinformatics tools offered an unprecedented opportunity to study viral evolution in the context of the population genetics and HLA driven selection pressures which must inevitably play an important role in determining the outcome of the infection. We focused on HLA i) since during CHIKV infection T cell responses are observed [38], and ii) it has been proposed that these strong responses, although not involved in the control of the virus during the acute phase of the primo-infection, might provide protection upon re-infection [39] and thus limit virus dissemination to the community. These strong T-cell responses might exert a potent selection on CHIKV evolution.

Using well-established predictive and analytical tools, we showed how amino acid substitutions may affect cell-mediated immune recognition. The existence of a continuous lineage from the Asian cluster, through the Indian Ocean cluster, and to the African cluster, reveal a remarkable correspondence between amino acid sequence variability and putative HLA class I-restricted recognition patterns. The influence of human host responses against CHIKV appears to play a dominant role in evolution. Our data showed that selective pressures on CHIKV are higher within individual countries than across countries (Figure 6 and Figure S2). Overall, this study confirms that HLA class I-restricted recognition patterns can be used as a base for studying the evolution of viruses [40], and in particular CHIKV. The growing capacity of full genome sequencing and improved epitope prediction methods provide a powerful new high-resolution tool for classification, and may serve as primary criteria for vaccine development.

## Materials and Methods

### CHIKV Data Collection

A total of 189 structural and 116 non-structural CHIKV sequences isolated from humans were extracted from National Center for Biotechnology Information (NCBI) GenBank [41] and SwissProt [42]. From these, 73,516 nonameric peptide sequences (46,913 non-structural peptides, 26,564 structural peptides) were generated and used for the current analysis.

### HLA Frequency Data Collection

HLA allele frequencies of Africa, Indian Ocean, Asia, Europe and USA were extracted from the Allele Frequency Database [43] and used for the current analysis.

### Protein Sequence Analyses

The ClustalX program [44] was used to align and to construct the phylogram of CHIKV proteomes. Bootstrap analysis was performed on 1000 replicates to ascertain support for the groupings in the tree. The Shannon entropy was used to assess the variability of CHIKV proteomes [45]. For a given alignment, the entropy of an amino acid position *H*(*x*) is defined as *H*(*x*) = −∑ *P*(*x*) log *P*(*x*) where *x* is one of 20 amino acid residue types. *P*(*x*), the probability of occurrence of *x*, is estimated by *f*(*x*), the frequency of the appearance of residue type within the alignment column *P*(*x*)≈*f*(*x*) = *N*(*x*)/*L* where *N*(*x*) is the number of appearances of amino acid residue *x*, and *L* is the length of the column.

### Nucleotide Sequence Analyses

Synonymous substitutions may be used as a molecular clock for studying the evolutionary time of highly conserved sequences [29]. In this study, the Nei and Gojobori's method [29], [30] was applied to calculate the rates of synonymous (dS) and non-synonymous (dN) substitutions in the sequences under study. The dN/dS ratio (*ω*) indicates the extent of evolutionary divergence of DNA sequences. *ω*>1 suggests positive (diversifying) selection, *ω*<1 suggests negative (purifying) selection, and *ω* = 1 indicates no selection.

### Antigenic Diversity Analyses

ntigenic diversity among CHIKV proteins was defined as the minimal set of unique HLA class I-restricted T-cell epitopes or immunological signatures encoded by all CHIKV sequences [46]. Computational prediction of T-cell epitopes that bind to 21 common HLA-A alleles (A*0101, *0201, *0202, *0203, *0206, *0301, *1101, *2301, *2402, *2403, *2601, *2902, *3001, *3002, *3101, *3201, *3301, *6801, *6802, *6901 and *8001) and 20 HLA-B alleles (B*0702, *0801, *1501, *1503, *1517, *1801, *2705, *3501, *4001, *4002, *4402, *4403, *4501, *4601, *4801, *5101, *5301, *5401, *5701 and *5801) was performed using two online computational systems available in the Immune Epitope Database Analysis Resource (IEDB-AR) [47], namely Average Relative Binding (ARB) matrix and Artificial Neural Network (ANN). The ARB system is based upon a matrix of coefficients derived from the association of each of the 20 amino acids at each possible position along the peptide sequence [48]. The ANN system predicts HLA-binding peptides using an input representation consisting of a combination of sparse encoding, BLOSUM encoding and inputs derived from hidden Markov models [49].

### Hierarchical Clustering

A hierarchical clustering technique using the agglomerative algorithm was used to assess the immunological signatures of CHIKV in different outbreak countries [50]. The distance between the immunological signatures was computed by the single-linkage method as implemented in MATLAB version 7.0.

## Supporting Information

Figure S1 Consequences of antigenic drift in a T cell epitope. (A) T cell epitope are peptides derived from pathogens or host proteins which bind HLA molecules. Peptides of 9 to 11 amino acids binds to HLA class I and longer peptides to HLA class II molecules. T cell epitope peptide forms a ternary complex with a MHC (HLA) molecule on the surface on an antigen presenting cell and the T cell receptor (TCR) on the surface on a T cells. Formation of this ternary complex is necessary for T cell activation and activity. Mutation in nucleotide(s) can lead to a change in amino acid in T cell epitope (variant peptide). This change will modify the interactions of this peptide sequence either with the HLA molecule or with the T cells. (B) Mutation leading to amino acid may prevent epitope (variant peptide) to bind to the HLA molecule, thus no T cells will be stimulated. (C) Mutations can also lead to change in amino acids (variant peptide) interacting with the TCR. In this case, the outcome of the T cell response may be different. In the first scenario, the mutation generates a variant peptide which cannot be recognized by the TCR. Thus no T cell will be stimulated. In the second scenario, the new variant peptide generated can be recognized by the TCR, but this interaction will generate partial response on T cell specific for the wild type peptide. Such variant are called antagonists or altered peptide ligands (APL). It has been shown that memory T cells specific for the wild type peptide respond poorly (decrease in proliferation, cytoxocity or in IFN-g secretion) after stimulation with antagonist peptides. All these mechanisms represent powerful evasion mechanism of the immune response.
(0.20 MB TIF)

Figure S2 Summary of the 41 HLA class I molecules investigated in this study. Populations with high frequencies of a given HLA are noted. Potential CD8+ T-cell epitopes are 9 amino acids long based on Average Relative Binding matrix and Artificial Neural Network derived from the Immune Epitope Database and Analysis Resource. The number of antigenic transitions for CHIKV epitopes from binding to non-binding for a given HLA molecule is also noted.
(0.08 MB DOC)

## References

[1] Robinson M (1955). An endemic of virus disease in southern province, Tanganyinka territory, in 1952–1953.. Trans Royal Soc Trop Med & Hyg.

[2] Lumsden WHR (1955). An endemic of virus disease in southern province, Tanganyinka territory, in 1952-1953.. Trans Royal Soc Trop Med & Hyg.

[3] Halstead SB, Scanlon JE, Umpaivit P, Udomsakdi S (1969a). Dengue and chikungunya virus infection in man in Thailand, 1962-1964.. Am J Trop Med Hyg.

[4] Halstead SB, Udomsakdi S, Scanlon JE, Rohitayodhin S (1969b). Dengue and chikungunya virus infection in man in Thailand, 1962-1964. V. Epidemiologic observations outside Bangkok.. Am J Trop Med Hyg.

[5] Rao TR (1966). Recent epidemics caused by chikungunya virus in India.. Sci Culture.

[6] Parola P, de Lamballerie X, Jourdan J, Rovery C, Vaillant V (2006). Novel Chikungunya virus variant in travelers returning from Indian Ocean islands.. Emerg Infect Dis.

[7] Pastorino B, Muyembe-Tamfum JJ, Bessaud M, Tock F, Tolou H (2004). Epidemic resurgence of Chikungunya virus in democratic Republic of the Congo: identification of a new central African strain.. J Med Virol.

[8] Laras K, Sukri NC, Larasati RP, Bangs MJ, Kosim R (2005). Tracking the re-emergence of epidemic chikungunya virus in Indonesia.. Trans R Soc Trop Med Hyg.

[9] Pialoux G, Gaüzère BA, Jauréguiberry S, Strobel M (2007). Chikungunya, an epidemic arbovirosis.. Lancet Infect Dis.

[10] AbuBakar S, Sam IC, Wong PF, MatRahim N, Hooi PS (2007). Reemergence of endemic Chikungunya, Malaysia.. Emerg Infect Dis.

[11] Ng LC, Tan LK, Tan CH, Tan SS, Hapuarachchi HC (2009). Entomologic and virologic investigation of Chikungunya, Singapore.. Emerg Infect Dis.

[12] WHO (2006). Wkly Epidemiol Rec.

[13] Schuffenecker I, Iteman I, Michault A, Murri S, Frangeul L (2006). Genome microevolution of Chikungunya viruses causing the Indian Ocean outbreak.. PLoS Med.

[14] Beltrame A, Angheben A, Bisoffi Z, Monteiro G, Marocco S (2007). Imported Chikungunya infection, Italy.. Emerg Infect Dis.

[15] Lakshmi V, Neeraja M, Subbalaxmi MV, Parida MM, Dash PK (2008). Clinical features and molecular diagnosis of Chikungunya fever from South India.. Clin Infect Dis.

[16] Borgherini G, Poubeau P, Staikowsky F, Lory M, Le Moullec N (2007). Outbreak of chikungunya on Reunion Island: early clinical and laboratory features in 157 adult patients.. Clin Infect Dis.

[17] Lemant J, Boisson V, Winer A, Thibault L, André H (2008). Serious acute chikungunya virus infection requiring intensive care during the reunion island outbreak in 2005-2006.. Crit Care Med.

[18] Tsetsarkin KA, Vanlandingham VL, McGee CE, Higgs S (2007). A Single Mutation in Chikungunya Virus Affects Vector Specificity and Epidemic Potential.. PLoS Path.

[19] Bouvier NM, Palese P (2008). The biology of influenza viruses.. Vaccine.

[20] Bhattacharya T, Daniels M, Heckerman D, Foley B, Frahm N (2007). Founder effects in the assessment of HIV polymorphisms and HLA allele associations.. Science.

[21] Parham P, Ohta T (1996). Population biology of antigen presentation by MHC class I molecules.. Science.

[22] Chevillon C, Briant L, Renaud F, Devaux C (2008). The Chikungunya threat: an ecological and evolutionary perspective.. Trends Microbiol.

[23] Udhayakumar V, Ongecha JM, Shi YP, Aidoo M, Orago AS (1997). Cytotoxic T cell reactivity and HLA-B35 binding of the variant Plasmodium falciparum circumsporozoite protein CD8+ CTL epitope in naturally exposed Kenyan adults.. Eur J Immunol.

[24] Klenerman P, Rowland-Jones S, McAdam S, Edwards J, Daenke S (1994). Cytotoxic T-cell activity antagonized by naturally occurring HIV-1 Gag variants.. Nature.

[25] Bertoletti A, Sette A, Chisari FV, Penna A, Levrero M (1994). Natural variants of cytotoxic epitopes are T-cell receptor antagonists for antiviral cytotoxic T cells.. Nature.

[26] Tsetsarkin KA, McGee CE, Volk SM, Vanlandingham DL, Weaver SC (2009). Epistatic roles of E2 glycoprotein mutations in adaption of Chikungunya virus to Aedes Albopictus and Ae. Aegypti mosquitoes.. PLoS ONE.

[27] Kowalzik S, Xuan NV, Weissbrich B, Scheiner B, Schied T (2008). Characterisation of a chikungunya virus from a German patient returning from Mauritius and development of a serological test.. Med Microbiol Immunol.

[28] Arankalle VA, Shrivastava S, Cherian S, Gunjikar RS, Walimbe AM (2007). Genetic divergence of Chikungunya viruses in India (1963-2006) with special reference to the 2005-2006 explosive epidemic.. J Gen Virol.

[29] Nei M, Gojobori T (1986). Simple methods for estimating the numbers of synonymous and nonsynonymous nucleotide substitutions.. Mol Biol Evol.

[30] Suzuki Y, Gojobori T (1999). A method for detecting positive selection at single amino acid sites.. Mol Biol Evol.

[31] Khan AH, Morita K, Parquet Md Mdel C, Hasebe F, Mathenge EG, Igarashi A (2002). Complete nucleotide sequence of chikungunya virus and evidence for an internal polyadenylation site.. J Gen Virol.

[32] Peters B, Sidney J, Bourne P, Bui HH, Buus S (2005). The immune epitope database and analysis resource: from vision to blueprint.. PLoS Biol.

[33] Khan AM, Heiny AT, Lee KX, Srinivasan KN, Tan TW (2006). Large-scale analysis of antigenic diversity of T-cell epitopes in dengue virus.. BMC Bioinformatics.

[34] Smith DJ, Lapedes AS, de Jong JC, Bestebroer TM, Rimmelzwaan GF (2004). Mapping the antigenic and genetic evolution of influenza virus.. Science.

[35] Halstead SB (1966). Mosquito-borne haemorrhagic fevers of South and South-East Asia.. Bull Wld Hlth Org.

[36] Surtees G, Simpsom DIH, Bowen ETL (1970). Ricefield development and arbovirus epidemiology, Kano plain, Kenya.. Trans R Soc Trop Med Hyg.

[37] Boorman JPT (1968). Isolations of the arboviruses in the Lagos area of Nigeria, and a survey of antibodies to them in man and animals.. Trans R Soc Trop Med Hyg.

[38] Jaffar-Bandjee MC, Das T, Hparau JJ, Trotot PK, Denizot M (2009). Chikungunya virus takes centre stage in virally induced arthritis: possible cellular and molecular mechanisms to pathogenesis.. Microbes Infect.

[39] Kam YW, Ong EKS, Renia L, Tong JC, Ng LFP (2009). Immunobiology of Chikungunya and implications for disease intervention.. Microbes Infect.

[40] de Campos-Lima PO, Levitsky V, Brooks J, Lee SP, Hu LF (1994). T cell responses and virus evolution: loss of HLA A11-restricted CTL epitopes in Epstein-Barr virus isolates from highly A11-positive populations by selective mutation of anchor residues.. J Exp Med.

[41] Benson DA, Karsch-Mizrachi I, Lipman DJ, Ostell J, Wheeler DL (2006). GenBank.. Nucleic Acids Res.

[42] Boeckmann B, Bairoch A, Apweiler R, Blatter MC, Estreicher A (2003). The SWISS-PROT protein knowledgebase and its supplement TrEMBL in 2003.. Nucleic Acids Res.

[43] Middleton D, Menchaca L, Rood H, Komerofsky R http://www.allelefrequencies.net.

[44] Thompson JD, Gibson TJ, Plewniak F, Jeanmougin F, Higgins DG (1997). The CLUSTAL_X windows interface: flexible strategies for multiple sequence alignment aided by quality analysis tools.. Nucleic Acids Res.

[45] Shannon CE (1950). Prediction and entropy of printed English.. Bell System Technical Journal.

[46] Dudley ME, Rosenberg SA (2003). Adoptive-cell-transfer therapy for the treatment of patients with cancer.. Nat Rev Cancer.

[47] Zhang Q, Wang P, Kim Y, Haste-Andersen P, Beaver J (2008). Immune epitope database analysis resource (IEDB-AR).. Nucleic Acids Res.

[48] Bui HH, Sidney J, Peters B, Sathiamurthy M, Sinichi A (2005). Automated generation and evaluation of specific MHC binding predictive tools: ARB matrix applications.. Immunogenetics.

[49] Nielsen M, Lundegaard C, Worning P, Lauemøller SL, Lamberth K (2003). Reliable prediction of T-cell epitopes using neural networks with novel sequence representations.. Protein Sci.

[50] Barnard JM, Downs GM (1992). Clustering of chemical structures on the basis of two-dimensional similarity measures.. J Chem Inf Comput Sci.

