# Supplementary figures and images for: HLA Class I Restriction as a Possible Driving Force for Chikungunya Evolution
Source: PLoS One. 2010 Feb 26;5(2):e9291. doi: 10.1371/journal.pone.0009291 (PMC2829075; doi:10.1371/journal.pone.0009291)

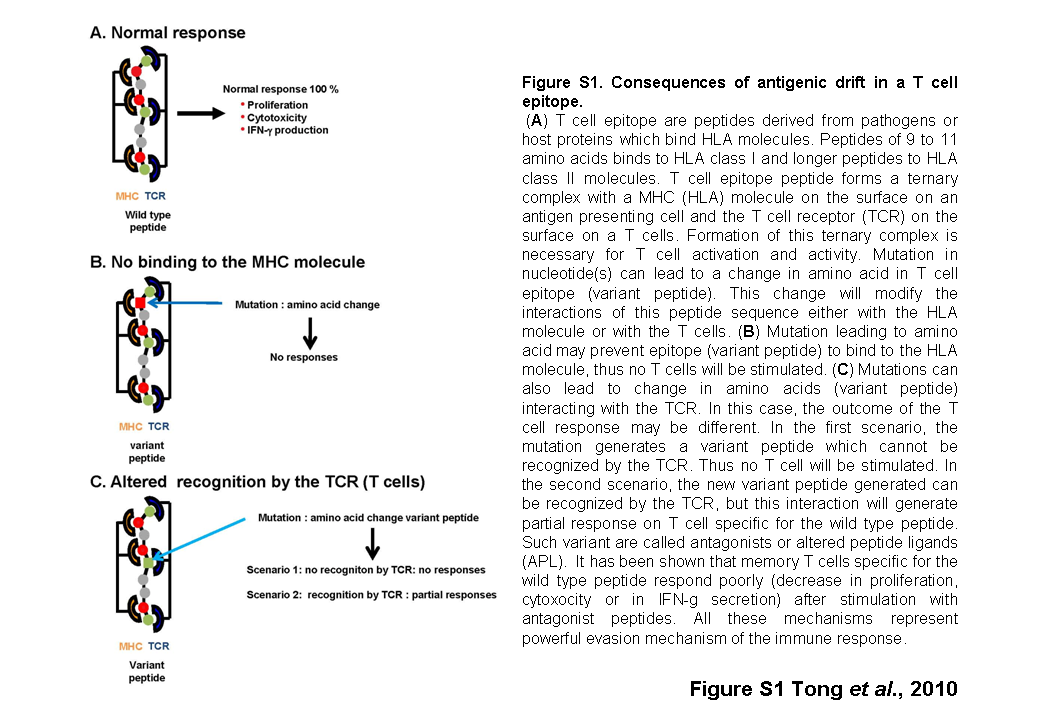

Supplement: Figure S1 — Consequences of antigenic drift in a T cell epitope. (A) T cell epitope are peptides derived from pathogens or host proteins which bind HLA molecules. Peptides of 9 to 11 amino acids binds to HLA class I and longer peptides to HLA class II molecules. T cell epitope peptide forms a ternary complex with a MHC (HLA) molecule on the surface on an antigen presenting cell and the T cell receptor (TCR) on the surface on a T cells. Formation of this ternary complex is necessary for T cell activation and activity. Mutation in nucleotide(s) can lead to a change in amino acid in T cell epitope (variant peptide). This change will modify the interactions of this peptide sequence either with the HLA molecule or with the T cells. (B) Mutation leading to amino acid may prevent epitope (variant peptide) to bind to the HLA molecule, thus no T cells will be stimulated. (C) Mutations can also lead to change in amino acids (variant peptide) interacting with the TCR. In this case, the outcome of the T cell response may be different. In the first scenario, the mutation generates a variant peptide which cannot be recognized by the TCR. Thus no T cell will be stimulated. In the second scenario, the new variant peptide generated can be recognized by the TCR, but this interaction will generate partial response on T cell specific for the wild type peptide. Such variant are called antagonists or altered peptide ligands (APL). It has been shown that memory T cells specific for the wild type peptide respond poorly (decrease in proliferation, cytoxocity or in IFN-g secretion) after stimulation with antagonist peptides. All these mechanisms represent powerful evasion mechanism of the immune response. (0.20 MB TIF) [file pone.0009291.s001.tif]
