# Supplementary material for: HLA Class I Restriction as a Possible Driving Force for Chikungunya Evolution
Source: PLoS One. 2010 Feb 26;5(2):e9291. doi: 10.1371/journal.pone.0009291 (PMC2829075; doi:10.1371/journal.pone.0009291)
Supplement: Figure S2 — Summary of the 41 HLA class I molecules investigated in this study. Populations with high frequencies of a given HLA are noted. Potential CD8+ T-cell epitopes are 9 amino acids long based on Average Relative Binding matrix and Artificial Neural Network derived from the Immune Epitope Database and Analysis Resource. The number of antigenic transitions for CHIKV epitopes from binding to non-binding for a given HLA molecule is also noted. (0.08 MB DOC) [file pone.0009291.s002.doc]

| **HLA** | **HLA-enriched populations** | **No. of potential epitopes** | **No. of antigenic transitions** |
| --- | --- | --- | --- |
| A*0101 | Europe | 705 | 51 |
| A*0201 | Africa, Asia, USA | 2399 | 160 |
| A*0202 | Africa, Asia, USA | 3203 | 136 |
| A*0203 | Africa, Asia, USA | 3373 | 141 |
| A*0206 | Africa, Asia, USA | 2779 | 158 |
| A*0301 | Asia, Europe | 1215 | 155 |
| A*1101 | Asia | 739 | 168 |
| A*2301 | Africa, Asia, Indian Ocean | 936 | 53 |
| A*2402 | Asia | 712 | 52 |
| A*2403 | Asia | 1223 | 181 |
| A*2601 | Asia | 651 | 143 |
| A*2902 | Africa, Europe | 1757 | 75 |
| A*3001 | Africa | 3680 | 172 |
| A*3002 | Africa | 1674 | 99 |
| A*3101 | Asia, Europe, USA | 2105 | 125 |
| A*3201 | Europe, Indian Ocean | 2332 | 149 |
| A*3301 | Africa, Asia | 341 | 372 |
| A*6801 | Africa, USA | 5721 | 196 |
| A*6802 | Africa, USA | 6744 | 185 |
| A*6901 | Africa | 5236 | 177 |
| A*8001 | Africa | 1557 | 71 |
| B*0702 | Europe, USA | 1397 | 164 |
| B*0801 | Europe | 1534 | 139 |
| B*1501 | Asia, Europe, USA | 3023 | 149 |
| B*1503 | Asia, Europe, USA | 6305 | 209 |
| B*1517 | Asia, Europe, USA | 3729 | 172 |
| B*1801 | Europe | 744 | 330 |
| B*2705 | Africa, Asia, Europe | 1156 | 171 |
| B*3501 | Africa | 3369 | 170 |
| B*4001 | Africa, Asia, Europe | 456 | 388 |
| B*4002 | Africa, Asia, Europe | 1797 | 188 |
| B*4402 | Europe | 476 | 376 |
| B*4403 | Europe | 904 | 192 |
| B*4501 |  | 1141 | 180 |
| B*4601 | Asia | 193 | 322 |
| B*4801 | Asia, USA | 399 | 358 |
| B*5101 | Africa, Asia, Europe, USA | 781 | 196 |
| B*5301 | Africa, USA | 800 | 180 |
| B*5401 | Asia, USA | 1348 | 102 |
| B*5701 | Africa, Asia, Europe | 1075 | 151 |
| B*5801 | Asia, Africa | 1109 | 154 |

**Figure S2**. **Summary of the 41 HLA class I molecules investigated in this study.**

Populations with high frequencies of a given HLA are noted. Potential CD8^+^ T-cell epitopes are 9 amino acids long based on Average Relative Binding matrix and Artificial Neural Network derived from the Immune Epitope Database and Analysis Resource. The number of antigenic transitions for CHIKV epitopes from binding to non-binding for a given HLA molecule is also noted.

**Figure S2 Tong et al., 2010**
